# SEER-based hypothesis-generating research for minimum lymph node evaluation in early-stage pancreatic ductal adenocarcinoma patients

**DOI:** 10.3389/fsurg.2025.1605726

**Published:** 2025-11-07

**Authors:** Heng Xu, Huaijuan Huang, Aimin Yan, Ruhang Li, Enfan Xiao, Hesong Wang, Yujie Wang, Taiyang Ge, Guangrui Huang

**Affiliations:** 1 School of Life Sciences, Beijing University of Chinese Medicine, Beijing, China; 2 School of Engineering Medicine, Beihang University, Beijing, China; 3 School of Chinese Materia Medica, Beijing University of Chinese Medicine, Beijing, China

**Keywords:** pancreatic cancer, invasive ductal carcinoma of the pancreas (IDCP), examined lymph nodes (ELNs), regional nodes positive (RNP), ELNs/RNP, optimal cut-off values for ELNs

## Abstract

**Background:**

Invasive ductal carcinoma of the pancreas (IDCP) is one of the most lethal of all solid cancers, with regional lymph nodes contributing to recurrent IDCP. Given the dismal prognosis of IDCP, the number of ELNs plays a vital role in patient prognosis. However, the optimal number of examined lymph nodes (ELNs) for stage I and II IDCP patients has not been defined by the 7th and 8th editions of the American Joint Committee on Cancer.

**Methods:**

All patients diagnosed with invasive ductal carcinoma pancreatic cancer were identified from the Surveillance, Epidemiology, and End Results (SEER) database (http://seer.cancer.gov/) using SEER*Stat Software (version 8.3.9.2). The minimum number of ELNs or ELN/regional nodes positive (RNP) ratio threshold for optimal survival of IDCP patients was calculated using the R packages “survminer” and “survival” and propensity score matching. Subgroup survival analysis based on the best cut-off values for ELNs was assessed for the following groups: age >69 years, age ≤69 years, female, male, N0, N1, T3, and stage I or II. We used a machine learning model (XGboost) to demonstrate that ELNs are the most significant prognostic factor in patients with IDCP. We also demonstrated significant prognostic effects and predictive models for the truncated values of ELNs using multivariate Cox regression. Finally, we assessed the correlation between ELN/RNP ratio and IDCP mortality using restricted cubic spline.

**Results:**

The present study demonstrates the following points: (1) ELNs are some of the most important factors affecting the prognosis of stage I and II IDCP patients. (2) The minimum cut-off value for stage I and II IDCP patients to achieve the best survival is ELNs ≥10, which is more suitable for surgical treatment options for stage II IDCP patients. (3) The optimal threshold of survival benefit for T3N1M0 patients is ELNs >12, with ELNs >7 for T3N0M0 patients. (4) Taking into consideration the effect of the number of RNP on the value of ELNs, the ELN/RNP ratio of 9 is the minimum threshold for optimal survival benefit in stage I or II IDCP patients.

**Conclusion:**

The minimum threshold for optimal survival of stage I or II IDCP patients in ELNs ≥10 and ELN/RNP ratio = 9, which is more appropriate for stage II IDCP patients. The optimal threshold of survival benefit for T3N1M0 patients is ELNs >12, with ELNs >7 for T3N0M0 patients.

## 1 Introduction

Pancreatic cancer is a leading cause of cancer-related death worldwide. The global burden of pancreatic cancer increased dramatically in the recent decade (1). The prevalence of pancreatic cancer in developed countries is higher than in developing countries. Regions of high prevalence include Europe, Australia, and North America (2). The risk factors for pancreatic cancer include cigarette smoking, diabetes mellitus, increased weight, alcohol consumption, and pancreatitis (3, 4, 36). Genetic factors also contribute to pancreatic cancer. Some of the high-risk inherited susceptibility genes are *BRCA2*, *CDKN2A*, *TP53*, and *MLH1* (5). In the early stage of pancreatic cancer, the symptoms are not obvious. Abdominal pain is a typical symptom in two-thirds of patients. Jaundice and weight loss are also symptoms of pancreatic cancer (6). However, pancreatic cancer typically has a very poor prognosis after diagnosis, with only 25% of patients surviving 1 year (7). The prognosis of patients with invasive ductal carcinoma of the pancreas (IDCP) is poor. IDCP is one of the most lethal malignancies of all solid cancers (8).

During the growth of IDCP, it often influences nearby tissue and organs such as the liver, lymph nodes, superior mesenteric artery, and portal vein (9). Recurrent carcinoma is responsible for the poor prognosis of IDCP after surgical resection. Regional lymph nodes, liver metastasis, and adjacent structures contribute to recurrent IDCP (10). Previous studies found that several factors were correlated with the prognosis of IDCP. Factors such as nerve invasion, tumor size, and tumor-associated macrophages (TAMs) were related with a poor prognosis of IDCP (11). Aggressive venous invasion was related with liver metastasis and has been considered a metastasis index of IDCP (9). In Pancreatic ductal adenocarcinoma (PDAC), better survival among N0 patients with increasing numbers of examined lymph nodes (ELNs) likely represents improved staging (12). A systematic review demonstrated that lymph node ratio and the number of positive nodes, but not the total number examined, are the factors associated with overall survival in PDAC (13). Hence, the number of ELNs is also an important prognostic factor for IDCP patients.

Given the dismal prognosis of IDCP, the impact of the number of ELNs on prognosis is particularly vital. However, no one has studied the minimum number of ELNs that would be of greatest benefit to stage I and II IDCP patients. In this study, we tried to investigate the relationship between ELNs and survival prognosis of stage I and II IDCP patients. In addition, we also investigated the optimal cut-off points to stratify postoperative prognosis of early-stage IDCP. First of all, we enrolled early-stage IDCP patients (stages I and II) from the Surveillance, Epidemiology, and End Results (SEER) database and evaluated the cut-off values of ELNs in the early stage of IDCP. Then, we conducted propensity score matching (PSM) analysis to calculate the cut-off value for ELNs. Different TNM classifications in stage I and II IDCP were evaluated. Furthermore, we used multivariate Cox regression analysis and a prediction model to evaluate the prognosis of early-stage IDCP patients. Ultimately, T3N0M0 and T3N1M0 IDCP patients were analyzed to determine the minimum ratio of ELNs to the number of positive lymph nodes [regional nodes positive (RNP)] that would confer the greatest benefit to the patient. This study provides novel insights into ELNs in terms of survival prognosis for stage I and II IDCP patients.

## 2 Methods

### 2.1 Patients

All patients diagnosed with invasive ductal carcinoma–type pancreatic cancer were identified from the SEER database (http://seer.cancer.gov/) using SEER*Stat Software (version 8.3.9.2). The SEER research data included SEER incidence and population data associated with age, sex, race, year of diagnosis, and geographic areas (including SEER registry and county). The clinical features included age, gender, tumor size, lymph nodes (LNs) examined, regional nodes positive, histological grade, histologic type, American Joint Committee on Cancer (AJCC) stage, total number of *in situ*/malignant tumors in a patient, TNM stage, primary tumor site, surgery, survival time, and survival status. Surgery is not effective in treating stage III or IV pancreatic cancer patients, so we only selected patients with stage I or II pancreatic cancer. Stage I and II patients who underwent surgery and had complete clinical information were chosen for further analysis.

### 2.2 Inclusion and exclusion criteria

The eligibility criteria for the study included the following: (1) patients with histological codes of 8500 and 8521, (2) patients subjected to the first surgical excision, (3) patients receiving lymph node dissection surgery, and (4) patients with a diagnosis of stage I or II pancreatic cancer. The exclusion criteria encompassed the following: (1) patients failing to record ELN and complete clinical information, (2) stage IIIB and IV patients due to surgery not being prioritized for them, (3) stage N2, N3, and M1 patients, and (4) patients of unknown or incomplete survival data and clinical features (unrecorded number of lymph nodes before preoperative examination and irradiation).

### 2.3 Calculation of the minimum ELN or ELN/RNP ratio threshold for the optimal survival of IDCP patients

In this study, the ELNs were divided into low and high subgroups, and an attempt was made to evaluate all possible divisions of the ELN data. The function “surv_cutpoint” from the R packages “survminer” and “survival” was utilized to discover the optimal cut-off value of ELNs. The cut-off point was defined as the value at which the survival prognosis of the two groups differs most significantly among all possible subgroups of ELNs. We collected the cut-off points of ELNs and the ELN/RNP ratio from patients with stage I and II IDCP. In addition, we analyzed the threshold of ELNs and the ELN/RNP ratio for patients at the T3N0M0 or T3N1M0 stage of the disease.

Before survival analysis, patients at stage I and II underwent PSM for the purpose of adjusting potential biases by selecting statistically different variables in the propensity model. A caliper—defined as the maximum tolerated difference between matched subjects in terms of “non-perfect” matching—was set at 0.01. The selected variables of stage I and II patients included age, gender, T-staging, N-staging, and stage of cancer. For these patients, the selected variables in the propensity model covered age, gender, radiation, and stage of cancer.

Finally, we assessed the correlation between the ELN/RNP ratio and IDCP mortality using restricted cubic spline (RCS).

### 2.4 Subgroup survival analysis of optimal cut-off values for ELNs

We had previously collected the optimal cut-off values of ELNs for survival of stage I and II patients. These data were subjected to subgroup survival analysis to evaluate the survival benefit of each group (age > 69 years, age ≤ 69 years, females, males, N0, N1, T1, T2, T3, stage I, and stage II).

### 2.5 Multivariate cox regression analysis and evaluation of model prediction efficiency

Based on the optimal cut-off values for ELNs associated with survival benefit, model 1 included clinical factors such as ELNs, age, sex, T-staging, and N-staging. Adjusted model 2 covered ELNs, age, gender, T-staging, N-staging, radiation, and chemotherapy.

The forest plot shows the Odds ratio (OR) and *P*-values for each clinical factor in this prediction model.

R software’s rms package generated an alignment diagram of a multivariate model, indicating the 1-, 2-, and 3-year survival rates of each subgroup. We used the C-index to quantify the discriminative ability of the nomogram, which estimated the difference between predicted and actual survival. We also performed a decision curve analysis (DCA) to evaluate the clinical benefit of our model.

### 2.6 Statistical analyses

SPSS (Version 26.0) and R 3.4.1 software (http://www.r-project.org) were employed to analyze the data. Pearson's chi-square test and the independent t-test were used to compare the baseline pathological characteristics. The Kaplan–Meier method was utilized to calculate the cumulative survival rate; a Student’s *t*-test was used for the logarithms to compare the survivorship curve. The prognostic factors that proved statistically significant in univariate analysis were analyzed via the multivariate Cox proportional hazard model. Results were measured with 95% confidence intervals (CI), and a two-tailed *P-*value of <0.05 was considered statistically significant.

## 3 Result

### 3.1 Identifying the minimum ELN threshold for the optimal survival of IDCP patients

**Figure 1:**
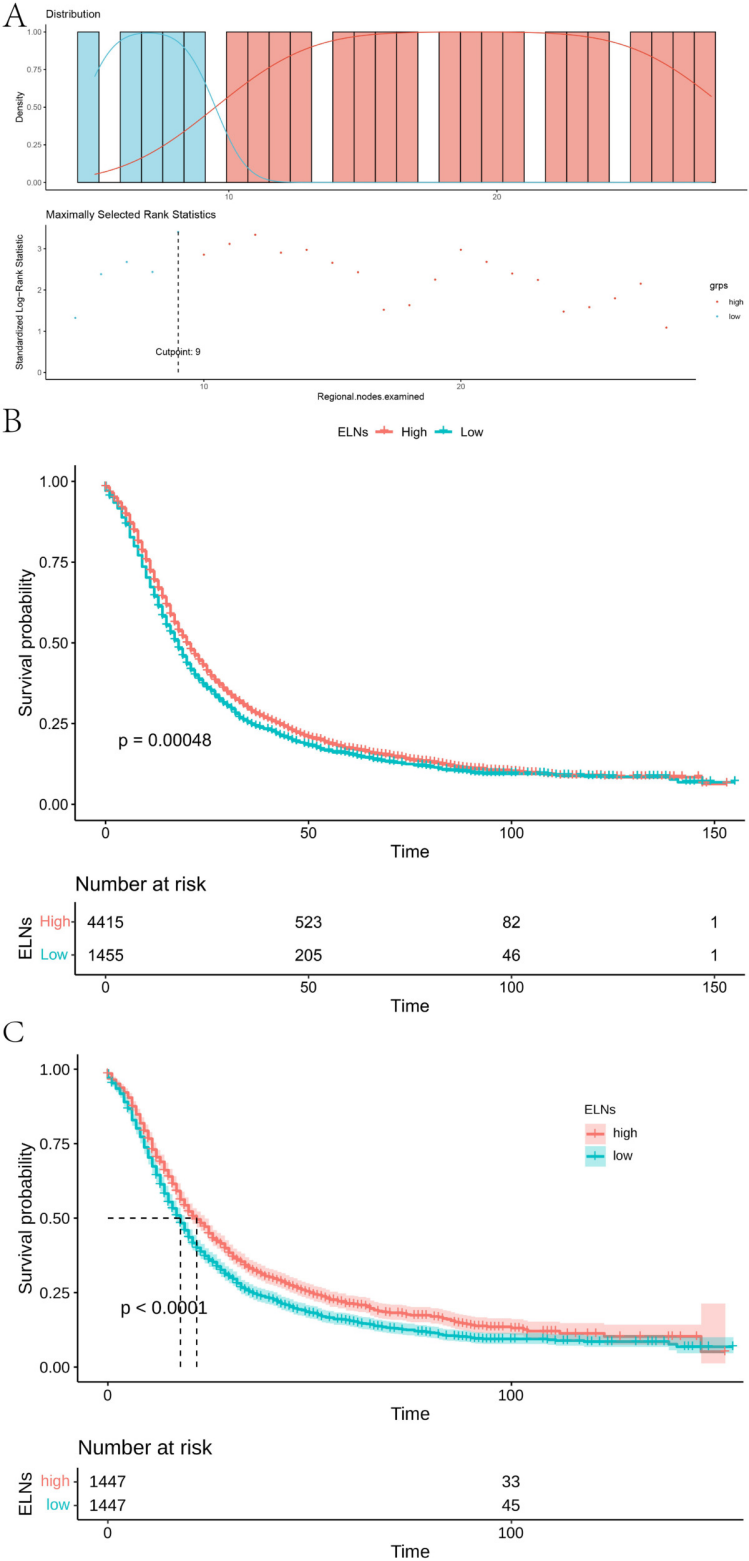
Optimal ELNs cut-off values for stage I and II IDCP patients. (**A**) Survival curve of high group (ELNs ≥ 10) and low group (ELNs < 10) [(best cut-off value of ELNs = 9; pre-PSM)]. (**B**) Survival curves of ELN-high group (ELNs ≥ 10) and ELN-low group (ELNs < 10) in all patients (pre-PSM). (**C**) Survival curves of ELN-high group and ELN-low group in all patients [best cut-off value of ELNs = 10; post-PSM)].

We assessed the correlation between the cut-off value of ELNs and the survival rate of IDCP patients using a method of exclusion. For ELN values of ≥7, there were significant survival differences between groups. For ELN values of ≥24, there were survival differences. Thus, 24 ≥ ELNs ≥ 6 was recommended to excise in clinical. When 10 was used as the divider, the two groups showed the most significant differences, with maximum chi-square values of 12.206, *P* = 0.00048 (Supplementary Table S1). Accordingly, ELN values of ≥10 were considered the minimum number for optimal survival for IDCP patients (Figures 1A,B). To test the sensitivity of the optimal cut-off value of ELNs, we randomly extracted 30%, 50%, and 80% of patients from the entire patient cohort. According to the results, the optimal cut-off value of ELNs was 9 (Supplementary Figure S1).

### 3.2 Baseline comparisons on ELNs ≥10 and <10 (pre-PSM and post-PSM)

To ensure the accuracy and reliability of results, we used a PSM analysis to reduce confounding factors; ELN values of >10 and<10 were analyzed as baseline values for each group. For pre-PSM patients, age (*P* = 0.005), T-stage (*P* < 0.001), N-stage (*P* < 0.001), and AJCC stage (*P* < 0.001) showed a maldistribution between groups (Supplementary Table S2). The maldistribution of influence factors between the two groups could potentially lead to bias in the survival analysis. For uneven baselines, the data were subjected to PSM to exclude influence factors. To guarantee the largest sample size on even baselines, our study used different caliper values and matching for data of different groups. To warrant equal numbers of patients in both groups, our study matched pancreatic cancer patients with ELN >10 and <10 in a 1:1 ratio. After PSM matching, there were no significant differences in age, T-stage, and N-stage between the two groups. As shown in Supplementary Table S3, a total of 1,447 pairs were successfully matched when the PSM model used 1:1 matching with 0.01 calipers. After matching, there were no statistical differences in the variables of age (*P* *=* 0.911), gender (*P* = 0.882), T-staging (*P* = 0.944), N-staging (*P* = 0.882), and AJCC stage (*P* *=* 1.000) between the two groups.

### 3.3 Post-PSM verification and subgroup analysis

**Figure 2:**
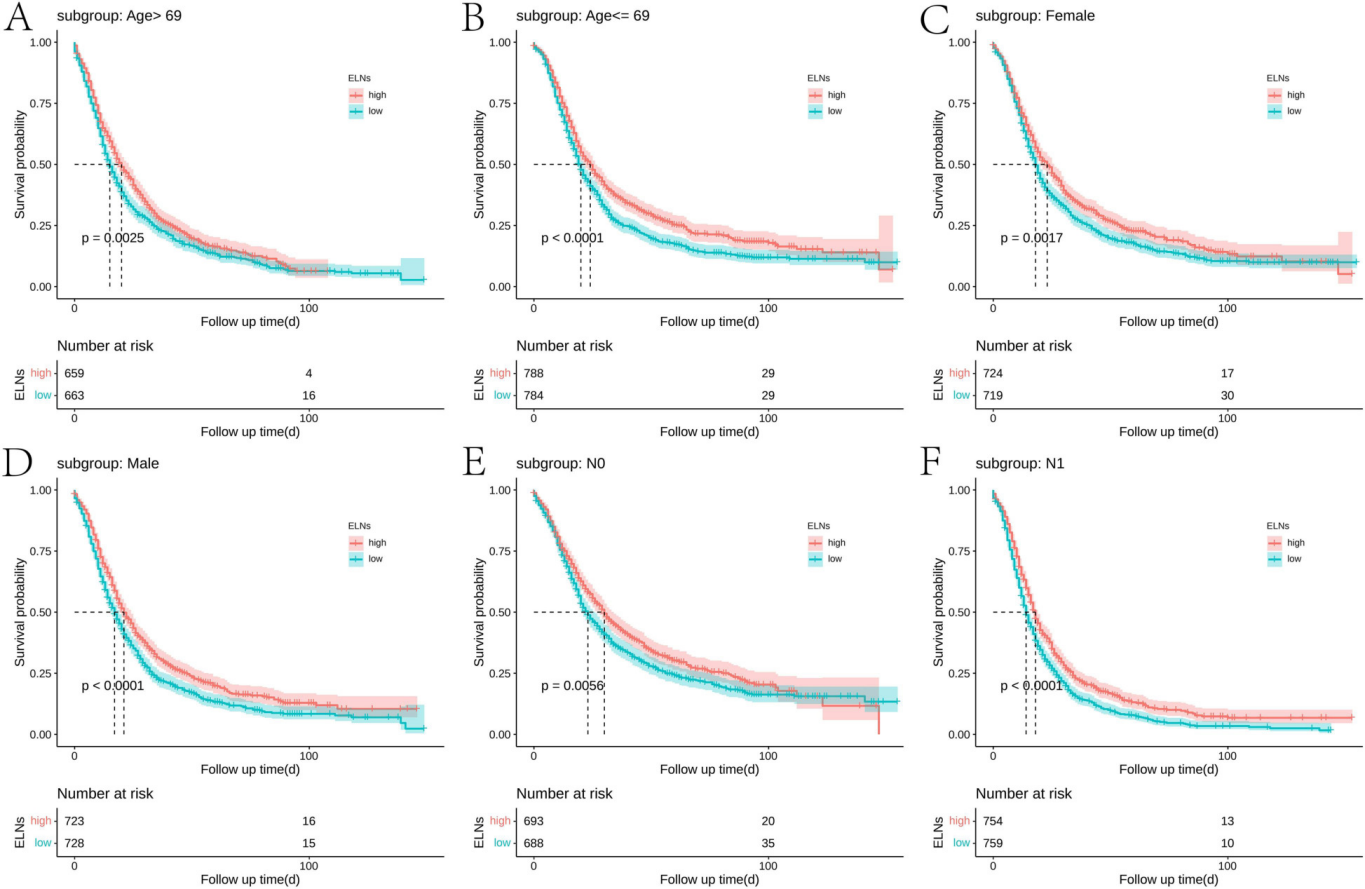
Survival curves of ELN-high group and ELN-low group in each subgroup: (**A**) age > 69 years, (**B**) age ≤ 69 years, (**C**) female, (**D**) male, (**E**) N0, and (**F**) N1 (best cut-off value of ELNs = 10; post-PSM).

According to the post-PSM results of IDCP patients, ELNs values of ≥10 and <10 were still important prognostic factors (Figure 1C). Then, we examined the effect of optimal cut-off values for ELNs on the prognosis of each subgroup of IDCP. According to the survival analysis for each subgroup (age > 69 years, age ≤ 69 years, female, male, N0, N1, T3, and stage II), patients with ELNs values of ≥10 had better estimated median survival rates (Figure 2, Supplementary Figure S2, Supplementary Table S4). However, no significant prognostic differences were found in the T1, T2, and stage I subgroups (Supplementary Figure S2). We also presented the estimated OR risk value for each subgroup based on the optimal cut-off point of ELNs in IDCP patients (post-PSM) (Supplementary Figure S3). ELN values of ≥10 remained beneficial for survival in stage T2 patients with OR >1 and *P*-values close to 0.05. Univariate and multivariate Cox regression analysis indicated that the ELN cut-off point of 10 was an independent prognostic factor (univariate Cox regression: HR, 1.241; 95% CI: 1.143–1.349; *P* < 0.001; multivariate Cox regression: HR, 1.263; 95% CI: 1.162–1.372; *P* < 0.001) (Supplementary Table S5).

### 3.4 The clinical prognostic model based on optimal cut-off value for ELNs

**Figure 3:**
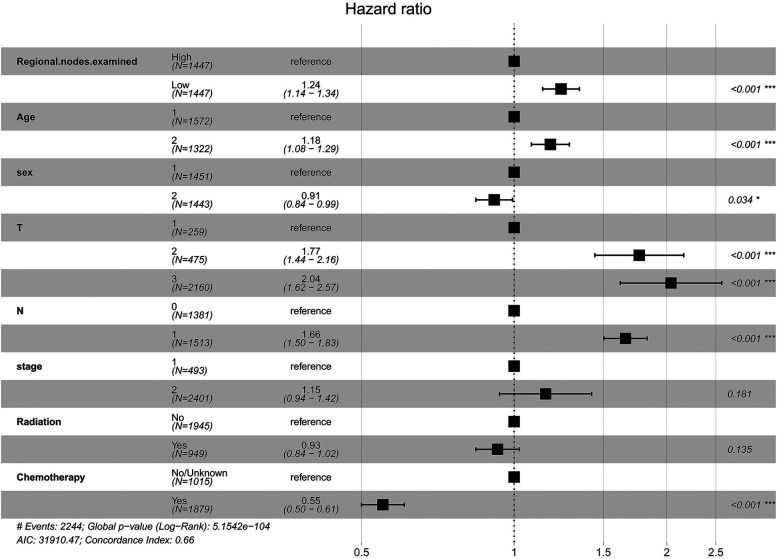
Forest plot of prognostic factors for IDCP patients (model 2: sex, the number 1 represents male and the number 2 represents female; age, the number 1 represents age ≤ 69 years and the number 2 represents age > 69 years).

A multivariate Cox regression prognostic model was constructed based on the optimal cut-off value for ELNs. Model 1 (base model) incorporated ELNs, age, sex, TNM stages, and stage of cancer. Model 2 (adjusted model) included ELNs, age, sex, stage, TNM stages, radiation, and chemotherapy. Figure 3 presented the estimated OR risk values for each factor, which indicated ELNs was a significant prognostic factor [OR = 1.24 (95% CI = 1.14–1.34), *P* < 0.001]. Supplementary Figure S4 shows the visualized multifactor Cox regression model with a C-index value of 0.6597. DCA showed that model 2 had a higher predictive efficacy than model 1, with better mortality prediction ability (Supplementary Figure S5).

### 3.5 XGBoost identifies ELNs as a significant prognostic factor in patients with early-stage IDCP

**Figure 4:**
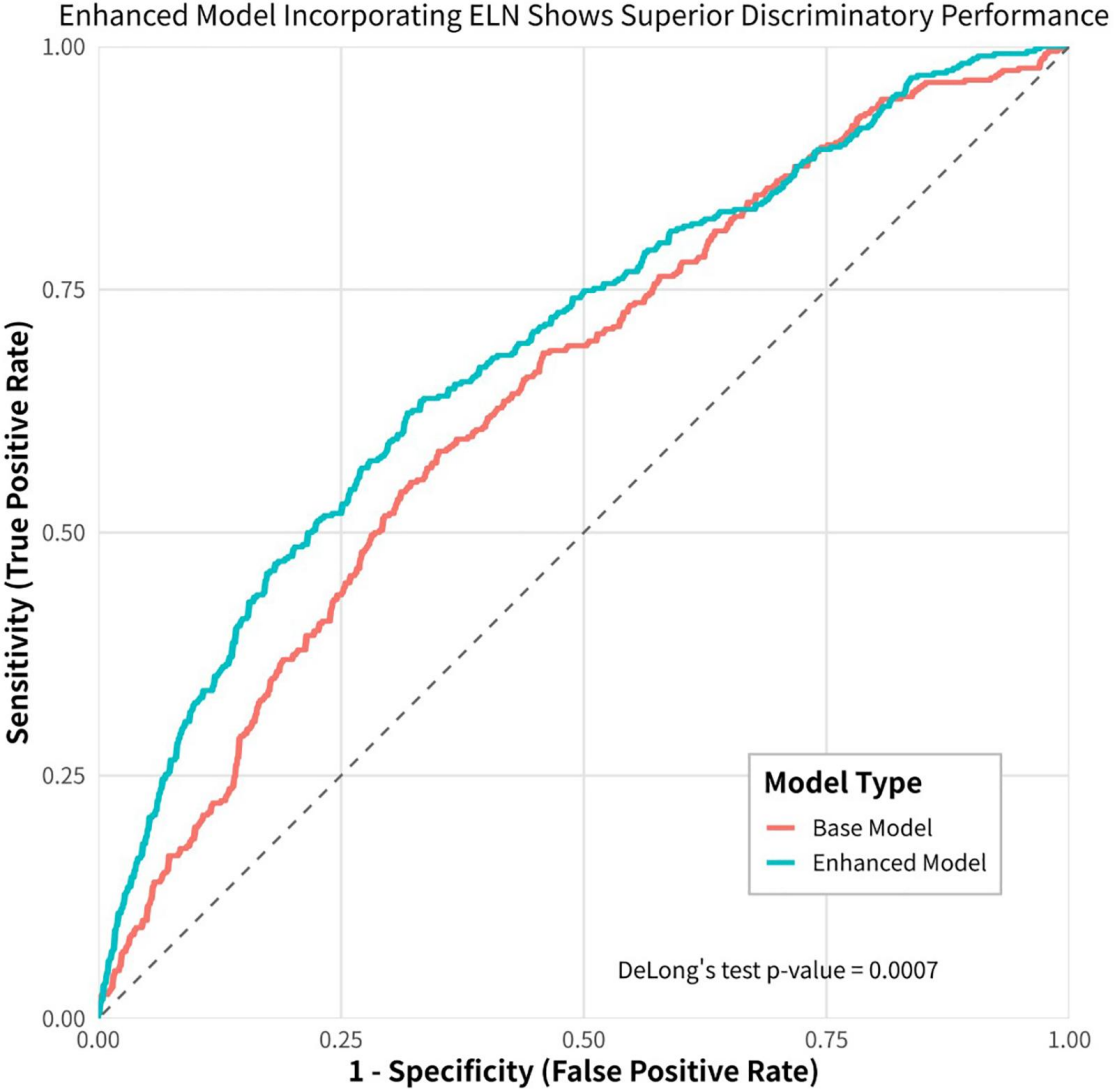
Machine learning (XGboost) model shows that ELN is the most important prognostic factor for early-stage IDCP patients.

We employed both baseline and adjusted XGBoost models to predict mortality in stage I and II IDCP patients. The baseline model incorporated the following variables: age, sex, surgery, tumor size, T-stage, N-stage, primary site labeled, regional nodes positive, and total number of *in situ*/malignant tumors per patient. The adjusted model included all variables from the baseline model, with the addition of regional nodes examined. The results demonstrated that the adjusted model exhibited higher sensitivity in predicting patient prognosis (*P* < 0.05) (Figure 4). The machine learning model (XGBoost) indicated that ELN risk categories serve as a significant prognostic factor for patients with IDCP.

### 3.6 ELN cut-off point for optimal survival benefit of T3N0M0 and T3N1M0 patients with IDCP

**Figure 5:**
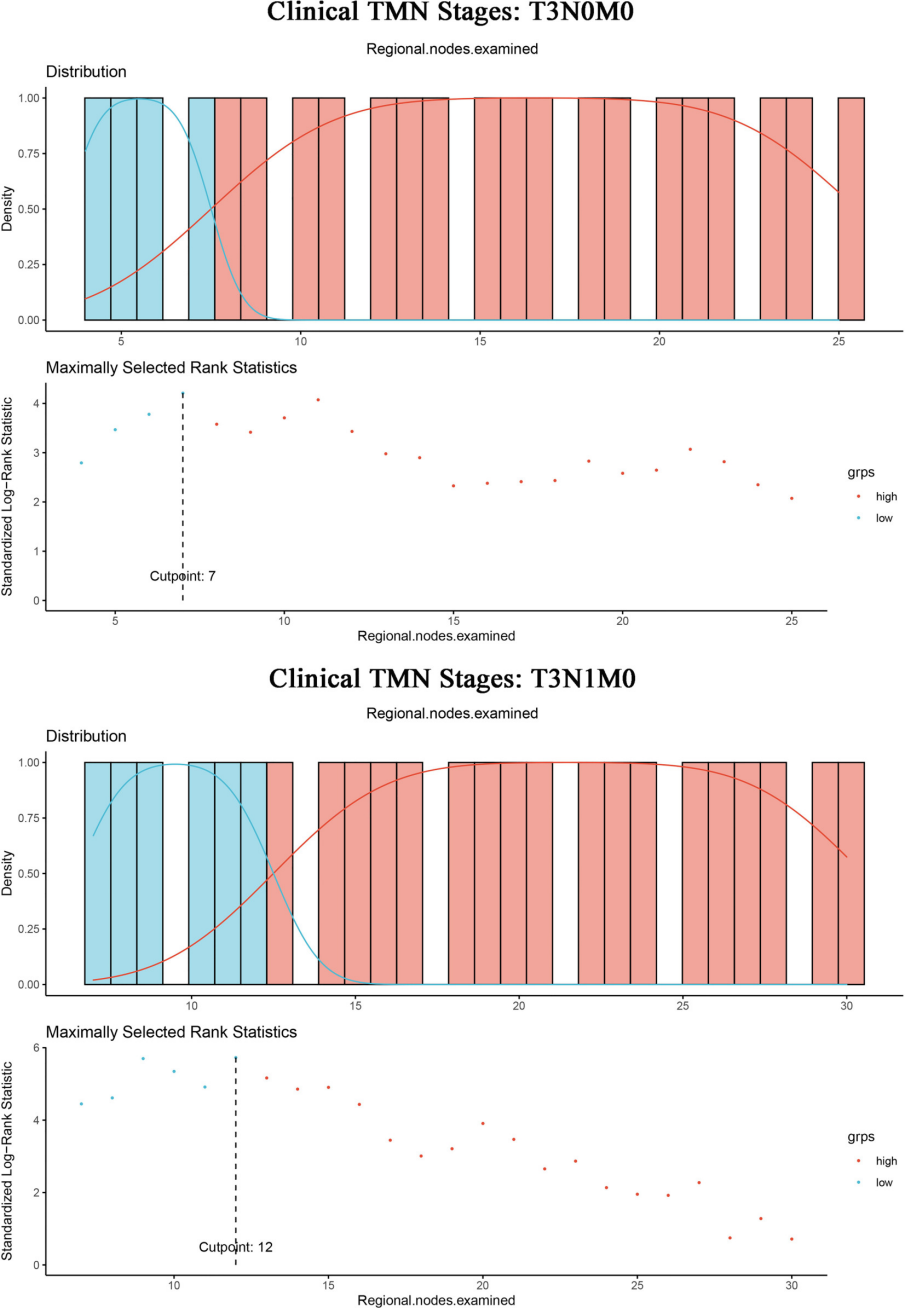
ELN cut-off point = 7 for optimal survival benefit of T3N0M0 patients with IDCP; ELN cut-off point = 12 for optimal survival benefit of T3N1M0 patients with IDCP.

In addition, given the small number of patients in T1 and T2 stages, further analysis of ELN cut-off values for optimal survival benefit was conducted only for patients in the T3 stage. We calculated the optimal cut-off point for T3N0M0 patients with IDCP. The result showed an ELN value of 7 was the most significant division point for the prognostic differences between the two groups (ELNs > 7 vs. ELNs ≤ 7) (Figure 5, Supplementary Figure S6). Likewise, the optimal cut-off point for patients with T3N1M0 IDCP was calculated. An ELN value of 12 was the most significant division point for the prognostic differences between the two groups (ELNs > 12 vs. ELNs ≤ 12).

### 3.7 XGBoost demonstrates that ELNs are the most important prognostic factor for T3N1M0 patients with IDCP

**Figure 6:**
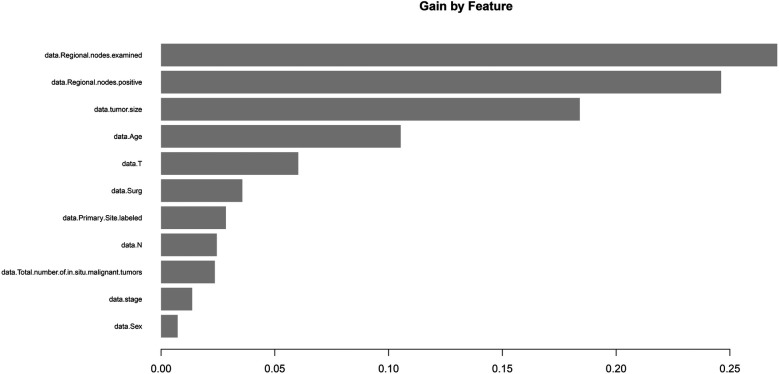
Machine learning (XGboost) model showed that ELN is the most important prognostic factor for T3N1M0 IDCP patients. ELNs, examined lymph nodes; RNP, regional nodes positive; IDCP, invasive ductal carcinoma of the pancreas.

Patients with T3N1M0 IDCP were selected as the target population, and the outcome variable was set to “death.” This XGboost model included the following variables: age, sex, surgery, tumor size, T-stage, N-stage, primary site labeled, regional nodes examined, regional nodes positive, and total number of *in situ*/malignant tumors for a patient. The model provided the following results: ELNs were the most significant predictor (Figure 6).

### 3.8 Identifying the minimum ELN/RNP ratio threshold for the optimal survival of IDCP patients

**Figure 7:**
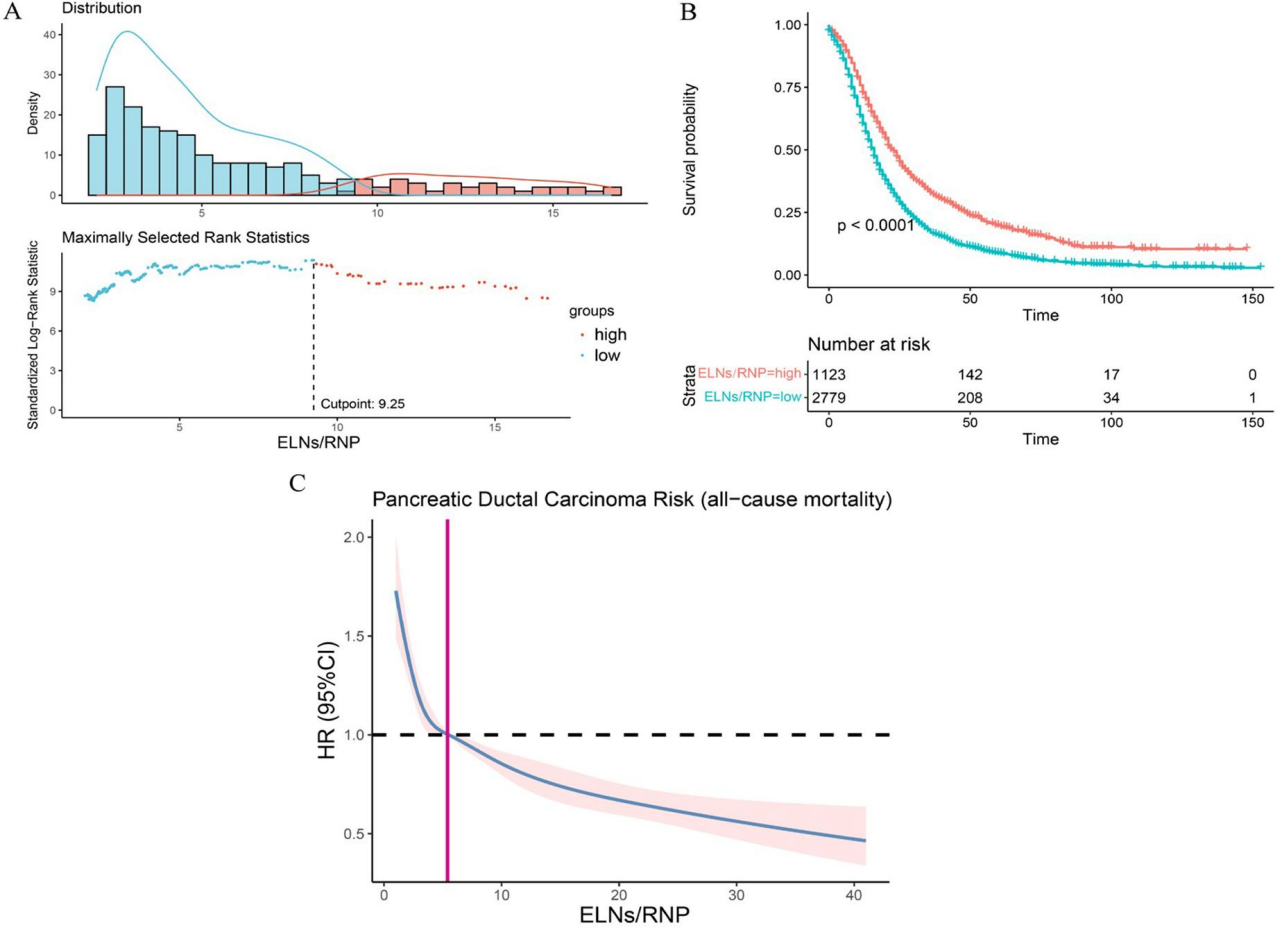
The evaluation of minimum ELN/RNP ratio. (**A**) ELN/RNP ratio = 9.25 is the threshold for survival benefit. (**B**) Survival curves of all-cause death in patients with a high ratio and low ratio of ELN to RNP. (**C**) Correlation between ELN/RNP ratio and all-cause mortality risk in IDCP stage I and II patients. ELNs, examined lymph nodes; RNP, regional nodes positive; IDCP, invasive ductal carcinoma of the pancreas.

As mentioned earlier in this study, the ELNs of IDCP are dependent on RNP, and thus the survival of pancreatic cancer patients is influenced by the ELN/RNP ratio. Therefore, we evaluated the minimum ratio of ELNs to RNP for optimal survival benefit of IDCP patients. As shown in Figure 1, an ELN/RNP ratio of 9.25 is the threshold for survival benefit in IDCP patients, with the risk of death due to IDCP gradually decreasing with an increasing ELN/RNP ratio (Figure 7). Therefore, an ELN/RNP ratio of 9 is the optimal cut-off value for clinical benefit.

## 4 Discussion

LN metastasis is an important prognosis factor for PDAC (14, 15). Previous studies have retrospectively indicated that the accuracy of TNM staging hinges on the number of ELNs and that the minimum number of ELNs should range between 12 and 17 (12, 16–19). Recent research has reported that 19 or more ELNs are required to ensure examination quality of LN in patients undergoing distal pancreatectomy (20). As the total number of ELNs increases, the likelihood of finding node-positive disease increases as well. A study found that the optimal threshold for the accuracy of AJCC staging system was 12 ELNs, although current guidelines recommend that at least 15 nodes be examined during pancreatoduodenectomy (21–23). IDCP is an aggressive subtype of pancreatic cancer. To date, the minimum number of ELNs that would be of greatest benefit to stage I and II IDCP patients has not been identified.

A clinically significant question is whether the required number of LN dissections can be evaluated in the majority of pancreatic cancer surgeries. An Italian study adopted the standard lymph node dissection guidelines as defined by the International Study Group on Pancreatic Surgery (ISGPS), with the average number of lymph nodes being 30.8 (24). A Japanese study showed that a median of 28 lymph nodes could be retrieved through careful pathological examination (25). Similarly, a study demonstrated that the median number of lymph nodes in standard LN dissection was 24, based on pancreatic cancer resection performed on a large number of patients at Heidelberg University Hospital (26). The relationship between the number of lymph node dissections and patient prognosis is a clinical issue worthy of further research, and it is also a significant method to improve patient survival rates. At present, numerous studies have focused on recommendations regarding the number of lymph node dissections for pancreatic cancer patients at all stages. However, for patients with early invasive pancreatic cancer, who received an early diagnosis but with tumors showing rapid malignant changes, there are no studies or recommendations for the number of lymph node dissections. Our study utilized surgical data of 5,870 stage I and II IDCP patients and identified that an ELN value of ≥10 is the minimum number for optimal survival benefit. To exclude confounding factors, such as age, gender, and disease severity, we performed validation on PSM-matched patients and found that ELN values of ≥10 remained a significant prognostic factor for stage I and II IDCP patients (*P* < 0.0001). Moreover, subgroup analysis showed that patients with ELN values of ≥10 had a better prognostic profile than those with ELN values of <10 in the subgroup with variables of age > 69 years, age ≤ 69 years, female, male, N0, N1, T3, and stage II; however, the T1, T2, and stage I patients did not show the same trend due to the small sample size.

Regarding the optimal number of LNs to examine, recommendations vary between 11 and 17 (27), with at least 15 often considered standard (22, 23). However, many proposed thresholds primarily aim to maximize prognostic differentiation between patient groups (19, 28). A notable update in the AJCC 8th edition of pancreatic cancer staging involves refining the nodal (N) classification (29). In particular, N1 now denotes tumors with 1–3 positive LNs, while N2 applies to tumors with ≥4 positive LNs. This revision underscores the critical role of LN status in pancreatic cancer staging (30, 31). Furthermore, tumor (T) stage is now defined solely by tumor size. However, in studies focusing on early invasive pancreatic cancer, ELNs ≥10 are still beneficial for survival in stage T2 patients with OR >1 and *P*-values close to 0.05. It is justified to infer that an ELN value of ≥10 is a more appropriate option for Stage II IDCP patients in surgery. Univariate and multifactorial Cox regression also showed that ELN values of ≥10 were significant prognostic factors. In addition, we independently analyzed the optimal ELN cut-off point for patients with T3N0M0 and T3N1M0 IDCP; ELN values of >7 were found to be the optimal threshold for survival benefit in T3N0M0 patients, and ELN values of >12 were the optimal threshold for survival benefit T3N1M0 patients. It has been recognized that survival of pancreatic cancer patients is influenced by the ELN/RNP ratio. Therefore, we evaluated the minimum ratio of ELNs to RNP for survival benefit in IDCP patients. Results revealed that an ELN/RNP ratio of 9 was the best cut-off point for survival benefit in IDCP patients; moreover, the ELN/RNP ratio is proportional to the mortality risk of IDCP.

Based on these findings, we propose that a minimum of 10 ELNs be examined and a ELN/RNP ratio of 9 be maintained as the cut-off value for stage I and II IDCP patients to achieve optimal survival; these values are more appropriate for stage II IDCP patients due to the limited number of stage I patients.

The relationship between ELN count and survival may follow a U-shaped curve, reflecting a balance between two opposing forces. Insufficient staging and residual disease risk (ELNs too low): An insufficient number of ELNs leads to a risk of under-staging (stage migration) and leaving behind metastatic deposits, which can become a source of recurrence. Immunological detriment (ELNs too high): On the other hand, excessively extensive lymphadenectomy might remove immunologically functional, non-metastatic lymph nodes. This could detrimentally impact the systemic anti-tumor immune response, particularly relevant in the era of adjuvant immunotherapy (32). The prognostic impact of ELN count is likely not merely anatomical but profoundly immunological. Tumor-draining lymph nodes (TDLNs) are pivotal hubs for antigen presentation, T-cell priming, and the generation of effector and memory T cells. Notch activation has been intimately linked to epithelial–mesenchymal transition (EMT), stemness, and lymphatic metastasis in PDAC and other cancers (33). For IDCP patients in whom the cancer has metastasized to other organs, lymph node dissection is an issue that must be addressed (34). Activation of Notch can enhance the invasive potential of tumor cells, facilitating their dissemination to regional lymph nodes. The net effect of lymphadenectomy on the systemic immune balance may depend on the relative distribution of immunosuppressive (e.g., PD-L1) and immunostimulatory niches across the nodal basin (35), a factor our dataset was unable to measure. The loss of these functional immune cell reservoirs through over-dissection could impair the efficacy of subsequent immunotherapeutic interventions.

## 5 Limits

Although our study rightly focused on classical ductal adenocarcinoma, surgical oncologists often confront sarcomatoid, acinar, or a mixed histology, which may have different patterns of spreading. We recommend that future multicenter collaborations explore optimal lymph node assessment in these subtypes.

## Data Availability

The original contributions presented in the study are included in the article/Supplementary Material, further inquiries can be directed to the corresponding author.
