# Supplementary material for: SEER-based hypothesis-generating research for minimum lymph node evaluation in early-stage pancreatic ductal adenocarcinoma patients
Source: Front Surg. 2025 Nov 7;12:1605726. doi: 10.3389/fsurg.2025.1605726 (PMC12634659; doi:10.3389/fsurg.2025.1605726)
Supplement: Supplementary file 1 [file Datasheet1.pdf]

Supplementary

Table S1. Analysis of the cut-off value for ELNs. (Log Rank (Mantel-Cox))

Cut-off value for ELNs	Chi-square score	P value
＜2 vs. ≥2	2.523	0.112
＜3 vs. ≥3	0.067	0.796
＜4 vs. ≥4	0.064	0.801
＜5 vs. ≥5	0.482	0.487
＜6 vs. ≥6	1.853	0.173
＜7 vs. ≥7	5.992	0.014
＜8 vs. ≥8	7.543	0.006
＜9 vs. ≥9	6.267	0.012
< 10 vs. ≥10	12.206	0.00048
＜11 vs.≥11	8.610	0.003
＜12 vs.≥12	10.245	0.001
＜13 vs.≥13	11.745	0.001
＜14 vs.≥14	8.954	0.003
＜15 vs.≥15	9.353	0.002
＜16 vs.≥16	7.594	0.006
＜17 vs.≥17	6.400	0.011
＜18 vs.≥18	2.635	0.105
＜19 vs.≥19	3.030	0.082
＜20 vs.≥20	5.563	0.018
＜21 vs.≥21	9.444	0.002
＜22 vs.≥22	7.718	0.005
＜23 vs.≥23	6.226	0.013
＜24 vs.≥24	5.362	0.021
＜25 vs.≥25	2.413	0.120
＜26 vs.≥26	2.711	0.100
＜27 vs.≥27	3.478	0.062

ELNs, examined lymph nodes.

Table S2. Baseline comparisons between groups (pre-PSM)

	**Nodes ≥ 10 (N = 4415)**	**Nodes < 10 (N = 1455)**	**P**
**Age**			**0.005**
≤ 69	2565 (58.1 %)	784 (53.9%)	
> 69	1850 (41.9 %)	671 (46.1%)	
**Sex**			0.568
Female	2234 (50.6%)	723 (49.7%)	
Male	2181 (49.4%)	732 (50.3%)	
**T**			< 0.001
T1	223 (5.05%)	136 (9.35%)	
T2	513 (11.6%)	242 (16.6%)	
T3	3679 (83.3%)	1077 (74.0%)	
**N**			< 0.001
N0	1226 (27.8%)	694 (47.7%)	
N1	3189 (72.2%)	761 (52.3%)	
**stage**			< 0.001
I	381 (8.63%)	253 (17.4%)	
II	4034 (91.4%)	1202 (82.6%)	

Table S3. Baseline comparisons between groups (post-PSM)

	**Nodes ≥ 10 (N = 1447)**	**Nodes < 10 (N = 1447)**	**P**
**Age**			0.911
≤69	784 (54.2 %)	788 (54.5%)	
>69	663 (45.8 %)	659 (45.5%)	
**Sex**			0.882
Female	719 (49.7%)	724 (50.0%)	
Male	728 (50.3%)	723 (50.0%)	
**T**			0.944
T1	132 (9.12%)	127 (8.78%)	
T2	238 (16.4%)	237 (16.4%)	
T3	1077 (74.4%)	1083 (74.8%)	
**N**			0.882
N0	688 (47.5%)	693 (47.9%)	
N1	759 (52.5%)	754 (52.1%)	
**stage**			1.000
I	247 (17.1%)	246 (17.0%)	

II	1200 (82.9%)	1201	(83.0%)

Table S4. Subgroup analysis for overall survival (post-PSM)

Characteristics	ELNs-High	ELNs-Low	subgroup analysis for overall survival	
			Hazard ratio (95% CI)	P value
**Age**				
≤69	788	784	1.266(1.150-1.395)	**5.68E-05**
>69	659	663	1.204(1.088-1.331)	**0.002**
**Gender**				
Male	723	728	1.280(1.160-1.410)	**3.23E-05**
Female	724	719	1.208(1.094-1.335)	**1.00E-03**
**T**				
T1	127	132	1.300(0.949-1.661)	0.1704
T2	237	238	1.2(1.023-1.450)	0.063
T3	1083	1077	1.3(1.160-1.358)	**2.14E-06**
**N**				
N0	693	688	1.198(1.07-1.33)	**0.005**
N1	754	759	1.307(1.192-1.433)	**1.52E-06**
**Stage**				
I	246	247	1.136(0.939-1.372)	2.68E-01
II	1201	1200	1.272(1.180-1.371)	**1.23E-07**

Table S5. Univariate and multivariate Cox regression analysis

Characteristics	Total(N)	Univariate analysis		Multivariate analysis	
		Hazard ratio (95% CI)	P value	Hazard ratio (95% CI)	P value
**Regional nodes examined**	2894				
High (ELNs ≥10)	1447	Reference			
Low (ELNs <10)	1447	1.241 (1.143-1.349)	**<0.001**	1.263 (1.162-1.372)	**<0.001**
**Age**	2894	1.286 (1.184-1.397)	**<0.001**	1.356 (1.247-1.474)	**<0.001**
**sex**	2894	0.909 (0.837-0.988)	**0.025**	0.940 (0.865-1.022)	0.145
**T**	2894	1.467 (1.367-1.575)	**<0.001**	1.300 (1.164-1.451)	**<0.001**
**N**	2894	1.750 (1.608-1.904)	**<0.001**	1.638 (1.487-1.805)	**<0.001**
**stage**	2894	1.979 (1.753-2.235)	**<0.001**	1.055 (0.858-1.296)	0.612

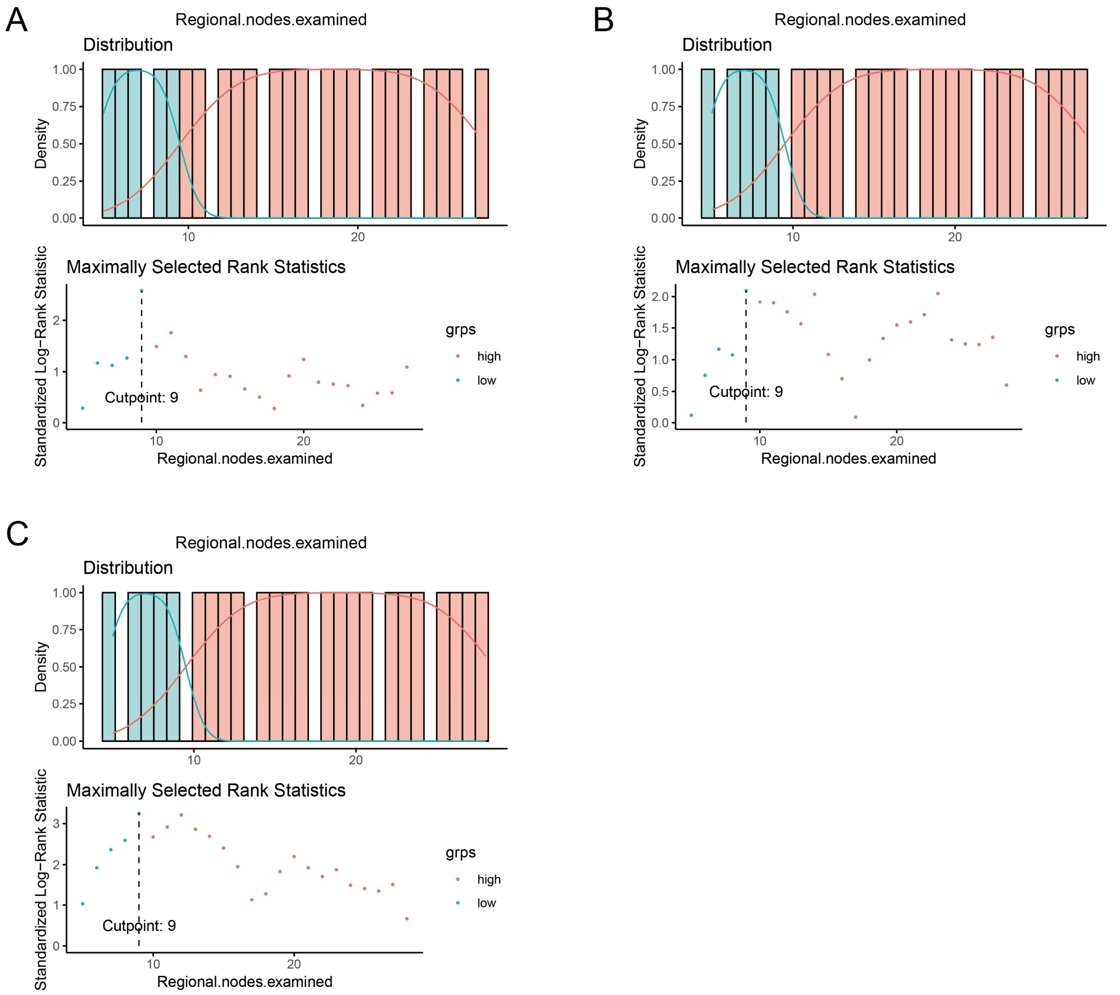

Figure S1. Sensitivity of the optimal cut-off value of examined lymph nodes (ELNs) in stage I and stage II IDCP patients. A. The optimal cut-off value of ELNs in 30% of patients. B. The optimal cut-off value of ELNs in 50% of patients. C. The optimal cut-off value of ELNs in 80% of patients.

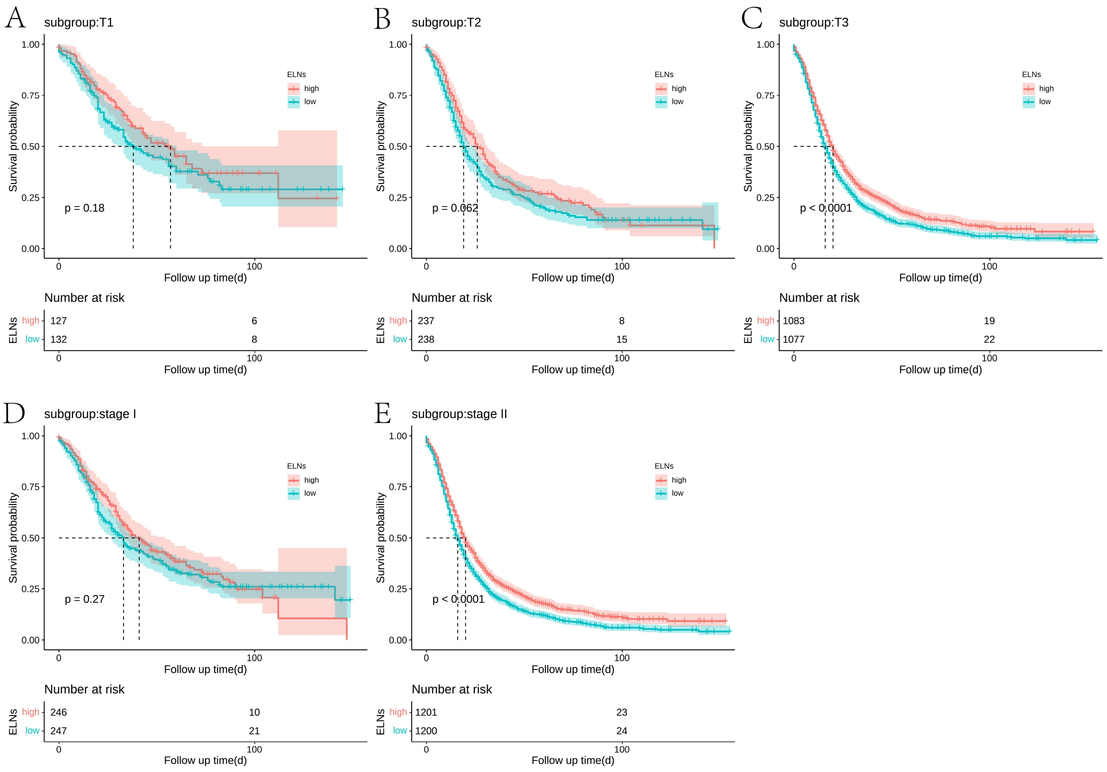

Figure S2. Survival curves of ELNs-high group and ELNs-low group in each subgroup ((A) T1, (B) T2, (C) T3, (D) stage I, (E) stage II) (post- PSM).

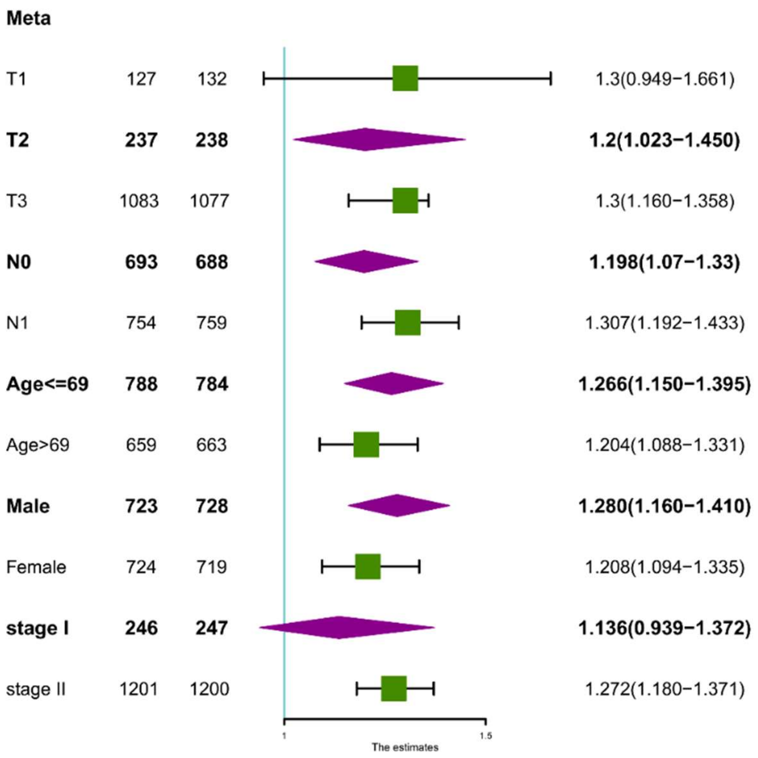

Figure S3. Risk value of each subgroup based on the optimal cut-off point of ELNs in IDCP patients (post-PSM).

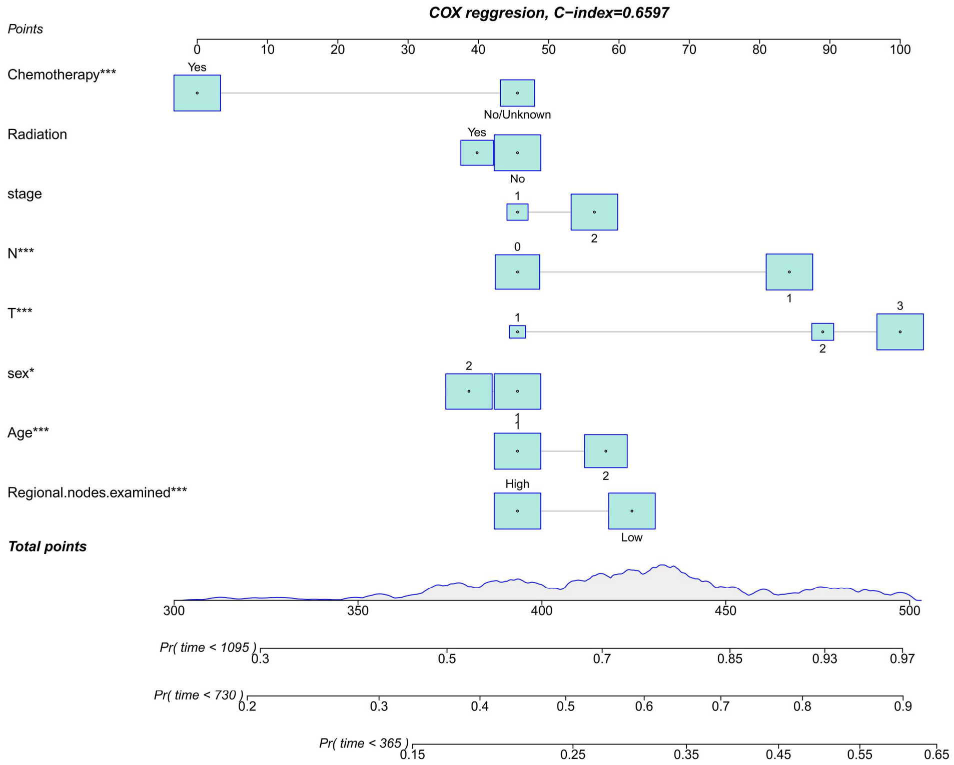

Figure S4. Multivariate Cox regression model for IDCP patients (model 2) (Sex, the number 1 represents male and the number 2 represents female; Age, the number 1 represents the age <= 69 and the number 2 represents the age > 69).

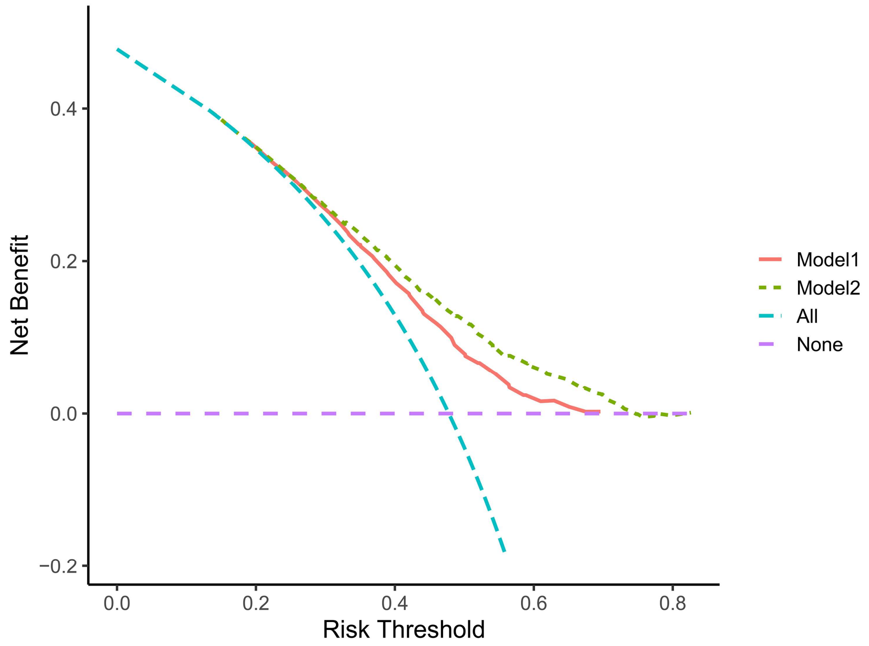

Figure S5. Decision curve analysis (DCA) of multivariate Cox regression model for IDCP patients.

(Model1=Regional nodes examined+Age+sex+T+N+stage; Model2=Regional nodes examined+Age+sex+T+N+stage+Radiation+Chemotherapy).

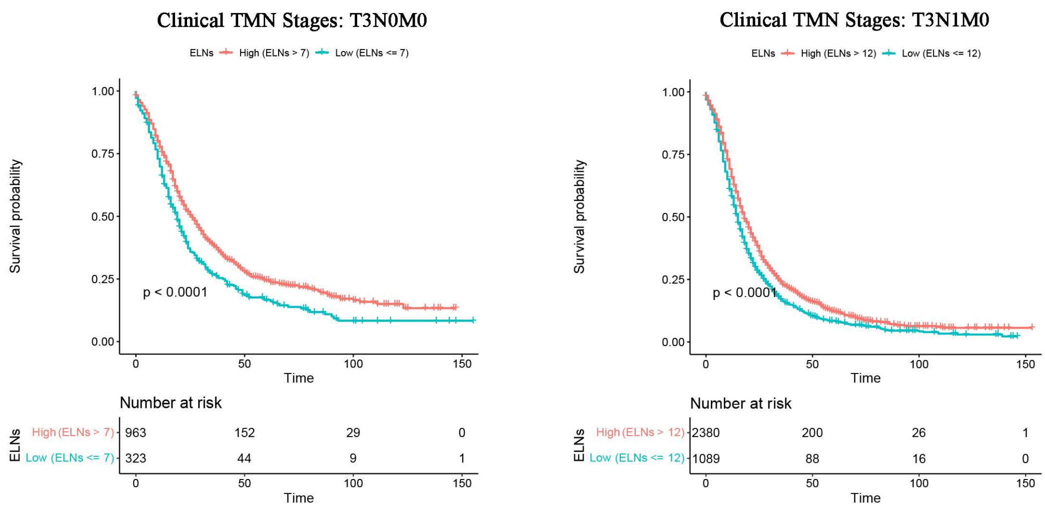

Figure S6. Survival curves of Stage T3N0M0 (The cutoff value of ELNs = 7) and T3N1M0 (The cutoff value of ELNs = 12).
